# Effects of adjuvants on IgG subclasses elicited by virus-like Particles

**DOI:** 10.1186/1479-5876-10-4

**Published:** 2012-01-05

**Authors:** Maria Luisa Visciano, Maria Tagliamonte, Maria Lina Tornesello, Franco M Buonaguro, Luigi Buonaguro

**Affiliations:** 1 Lab. of Molecular Biology and Viral Oncogenesis, Istituto Nazionale Tumori "Fond. G. Pascale", Naples-Italy

## Abstract

**Background:**

Virus-Like Particles (VLPs) represent an efficient strategy to present and deliver conformational antigens to the immune system, inducing both arms of the adaptive immune response. Moreover, their particulate structure surrounded by cell membrane provides an adjuvanted effect to VLP-based immunizations. In the present study, the elicitation of different patterns of IgG subclasses by VLPs, administered in CpG ODN1826 or poly(I:C) adjuvants, has been evaluated in an animal model.

**Results:**

Adjuvanted VLPs elicited a higher titer of total specific IgG compared to VLPs alone. Furthermore, while VLPs alone induced a balanced T_H_2 pattern, VLPs formulated with either adjuvant elicited a T_H_1-biased IgG subclasses (IgG2a and IgG3), with poly(I:C) more potent than CpG ODN1826.

**Conclusions:**

The results confirmed that adjuvants efficiently improve antigen immunogenicity and represent a suitable strategy to skew the adaptive immune response toward the differentiation of the desired T helper subset, also using VLPs as antigen.

## Introduction

The development of a safe and effective HIV-1 vaccine, either prophylactic or therapeutic, remains a major concern and a high priority for the scientific community. In this respect, given that strategies based on attenuated or inactivated pathogens are not suitable as HIV vaccines for safety reasons, alternative effective vaccination strategies are developed and evaluated [1].

In this framework, Virus-Like Particles represent a highly attractive type of subunit vaccine since they are self-assembling, non-replicating, non-pathogenic particles, similar in size and conformation to intact virions [2]. Moreover, VLPs can enter both MHC class I and class II antigen processing pathways in antigen presenting cells [3,4], eliciting both humoral and cellular immune responses [5]. VLP vaccines have been developed and licensed for HBV as well as HPV and immunological studies have showed induction of protective humoral and cellular immunity [6-8]. Additional VLP based vaccine for other viruses such as HCV, influenza virus and HIV-1 are in different stages of pre-clinical and clinical trial [9-15].

Human IgG consists of four subclasses contributing in different ways to humoral immunity against pathogens. Individual subclasses are elicited by different type of antigens: antibody responses to viral and bacterial protein antigens are mainly restricted to IgG1 and IgG3 [16-19], while IgG2 is generally produced in response to carbohydrate antigens [20-22]. In this regards, subjects with decreased levels of IgG2 antibodies show poor antibody responses to polysaccharide vaccines [23], suggesting this subclass is responsible for protection against encapsulated pathogens. In addition, several factors, including the structure and quantity of antigen [24] as well as the route and duration of antigenic stimulation may affect the subclass of IgG antibody produced. In particular, IgG1 and IgG3 subclasses mediate important protective, biological functions such as complement fixation, opsonization and induction of ADCC by NK cells [25,26].

Mice, similarly to humans, show four different classes of IgGs, named IgG1, IgG2a, IgG2b and IgG3, which functionally correspond to the human IgG1, IgG2, IgG4 and IgG3, respectively. Nevertheless, differences can be observed among the two animal species in the IgG subclasses to bind FcR, to fix complement or to undergo to cytokine-induced subclass switching [27]. Despite these differences, the overall structure of the humoral IgG pattern in mice and humans can be considered quite similar. In general, it is possible to conclude that in mice and humans IgG1 (as well as IgG4 in humans) is associated with a T_h_2 profile and the other subclasses are mainly associated with a T_h_1 profile [28].

As for other infectious diseases, the pattern of IgG subclass has been shown to play a role in the course of HIV infection and it has been reported to vary with progression status. Subjects enrolled in the French Asymptomatic Long-Term (ALT) cohort showed a strong IgG1 responses to Env and Pol antigens as well as a broad IgG subclass response to p24 [29]. Further studies have shown that anti-HIV humoral response characterized by a broad spectrum of IgG subclasses is associated with a non-progressor status [28,30].

Such observation strongly suggest that, in order to be protective, a HIV vaccine should elicit a broad and balanced IgG subclass immune response. So far all the soluble gp120 glycoprotein based HIV-1 vaccines have shown to elicit mainly IgG1 subclass antibodies both in humans and mouse, indicating a T_H_2 skewed response [31-34], and strategies to switch to a broader IgG subclass profile have been proposed [32,34]. Alternative vaccine approaches have been shown to induce a more balanced T_H_1 and T_H_2 response [35] or a T_H_1 skewed response [36,37]. In particular, adjuvants used in the vaccine formulation may influence the IgG profile, with CpG and MPL inducing a T_H_1 skewed immune response [38-42] while Al(OH)_3 _a T_H_2 profile [43,44].

Virus-like particles (VLPs) developed in our laboratory are based on the HIV-1 Pr55gag precursor protein (HIV-VLPs) and display a trimeric gp140 molecule from an Ugandan HIV-1 isolate of the A clade [45-48]. Such HIV-VLPs have shown to induce HIV-1-specific CD4+ and CD8+T cell responses as well as cross-clade neutralizing antibodies in immunized Balb/c mice [49,50]. Moreover, the intraperitoneal and intranasal administration of HIV-VLPs in mice have demonstrated to induce antibody responses at systemic as well as mucosal (vaginal and intestinal) levels [13,14]. Furthermore, we have previously reported that baculovirus-expressed HIV-1 VLPs developed in our laboratory induce secretion of both T_H_1 and T_H_2 cytokines in Monocyte-Derived Dendritic Cells (MDDC) [51,52] as well as in PBMCs [53,54].

In order to evaluate whether the pattern of IgG subclasses induced by baculovirus-expressed HIV-VLPs could be broaden and/or skewed toward a more pronounced T_H_1 profile, immunogenicity studies were performed using the two T_H_1-skewing adjuvants, CpG ODN1826 and poly(I:C) [55-57]. In particular, polyI:C is a synthetic double stranded RNA that potently induces IL-12 and type I IFNs through activation of innate immunity via endosomally expressed TLR3 and the cytoplasmic receptor MDA-5 [58,59]. Induction of type I IFNs results into DC maturation and B cell activation, followed by initiation of potent CD4+T cell and humoral immune responses, as shown in mice [60,61] and in non-human primates [42,62]. Furthermore, type I IFNs have been shown to induce, in a mouse model, cross presentation of proteins to generate CD8+ T cell responses [63,64].

CpG ODNs are synthetic oligonucleotides containing unmethylated CpG dinucleotides with specific motifs recognized by the innate immune system of vertebrates [65]. These motifs represent the ligand for TLR-9 expressed intracellularly in phagocytic cells [66] and can induce maturation, differentiation and proliferation of different immune cell types [67-71]. Upon induction, immune cells secrete cytokines and chemokines that create a T_H_1 biased immune environment [72-75]. Vaccines formulated with CpG motifs have been shown to elicite both cellular and humoral response [76-78].

The results in the present study show that both adjuvants, and more potently poly(I:C), are able to skew the immune response to HIV-VLPs toward a T_H_1 profile.

## Material and methods

### VLPs preparation

HIV-1 Virus-like Particles expressing on their surface modified HIV gp140 Clade A were prepared as previously described [79]. Briefly, HighFive insect cells, derived from Trichoplusia ni egg cell homogenates (Invitrogen Inc.), were propagated in SF900 serum-free medium supplemented with 1% antibiotics (Gibco-BRL) and 9 × 10^5 ^cells seeded in a 6-well plate were transfected with 10-20 μg of recombinant bacmid DNA, by the Cellfectin method (Gibco-BRL). After 72 hr incubation, recombinant baculovirus released in the supernatant of transfected cells (Passage 1) was used to infect 2 × 10^6 ^HighFive cells/well and the released recombinant baculovirus was collected after 72 hr incubation (Passage 2). A further infection step was performed to obtain the Passage 3 recombinant baculovirus to be used for large-scale VLPs preparation. HighFive cells were propagated in suspension and 4 × 10^9 ^cells were infected with the P3 recombinant baculovirus at a multiplicity of infection (M.O.I.) of 5 in a final volume of 100 ml. After 5 hr incubation in orbital shaker, cells were diluted with SF900 culture medium to a concentration of 1 × 10^6^/ml and incubated for 96 hrs in orbital shaker. Subsequently, supernatants were clarified by centrifugation at 2,000 g for 15 min at 4°C and VLPs were pelleted by ultra-centrifugation at 100,000 g for 75 min through a 25% sucrose cushion, as previously described [47,80] and resuspended in TNE buffer (10 mM Tris-HCl, pH 8.0, 100 mM NaCl and 1 mM EDTA).

### Immunization study

For the immunization study, 6 female Balb/c mice of 6-8 weeks old were immunized subcutaneously with 20 μg of HIV-VLP in combination or not with 20 μg of CpG ODN1826 or 100 μg of poly(I:C) (both from InvivoGen) as adjuvants. Mice received a priming and two homologous boosts, three weeks apart from each other. Blood samples were collected with retro-orbital puncture a week after each injection; sera were heat inactivated and stored at -80°C until used. Animal experiments were carried out following internationally recognized guidelines and were approved by the Italian Ministry of Health.

### ELISA

All ELISA determinations individually described below were developed with the following procedure. Positive reactions were visualized with TMB Ultra 1-step solution (Thermo Scientific) and stopped with 2N sulfuric acid. Absorbance was determined at O.D.450 nm and reactions were considered positive when exceeding the mean absorbance +3 standard deviations of equal dilutions of pre-immunization sera collected from animals. The antibody (Ab) levels were evaluated as the geometric mean titer of the last positive dilution of sera from the animals of each set. In all ELISA pre-immunization sera have been used as negative control.

### Measurement of specific anti-HIV IgG antibodies in mouse serum

The level of IgG antibodies in sera of immunized mice was determined by ELISA. Briefly 96-well MICROTEST assay plates (Becton Dickinson) were coated with 100 ng/well of B clade p24 **(**NIBSC-CFAR, Catalog #EVA620) or homologous A clade trimeric gp140 and incubated overnight at 4°C. Three-fold dilutions of mouse sera starting at a 1:100 dilution, were added to each well and incubated 2 hrs at 37°C. Horseradish peroxidase (HRP)-conjugated goat anti-mouse IgG (Thermo Scientific) was added to each well at a concentration of 1 μg/mL (100 ng) and incubated 2 hrs at 37°C.

### Determination of anti-gp140 and anti-p24 specific IgG subclasses

Specific anti-gp140 and anti-p24 IgG subclasses were determined by ELISA in sera of mice immunized as previously described. Specific HRP-conjugated goat anti-mouse IgG_1 _or biotin-conjugated goat anti-mouse IgG_2a_, IgG_2b _and IgG_3 _(all from Southern Biotech) were added individually to each well and incubated for 2 hr at 37°C. The anti mouse IgG_1 _was used at a 1:2,000 dilution. Alternatively, the biotin-conjugated goat anti-mouse IgG_2a_, IgG_2b _and IgG_3 _were used at a 1:5,000 dilution and a third HRP-conjugated anti-biotin Ab (Cell Signaling) was added to each well at 1:1,000 dilution for an additional 1 hr incubation at 37°C.

### Overtime evaluation of anti-gp140 and anti-p24 specific humoral immune response

The trend of the humoral immune response during the immunization protocol was evaluated. At each time point, three-fold dilutions of pooled sera from immunization groups were added to each well coated with recombinant gp140 or p24. Specific anti-mouse HRP-conjugated IgG was then added at 1 μg/mL concentration and plates were incubated for 2 hours a 37°C.

### T_H_1: T_H_2 index calculation

To determine whether the addition of adjuvants in the immunization protocol induced a T_H_1 (IgG2a and IgG3) or T_H_2 (IgG1) polarization, we proceeded as follows. The reciprocal value of the titer at last positive dilution for each IgG subclass was considered for each immunization protocol. The T_H_1:T_H_2 index was then calculated as ([IgG2a+IgG3]/2)/(IgG1). According to such calculation, an index value < 1 stands for a T_H_2 polarization; an index value > 1 stands for a T_H_1 polarization.

### Statistical analysis

Intergroup comparisons were performed with the unpaired two-sided Student's t-test. All P values were two-tailed and considered significant if less than 0.05.

## Results

### Immunization with HIV-1 VLP induces specific antibodies to HIV-1 antigens

**Figure 1:**
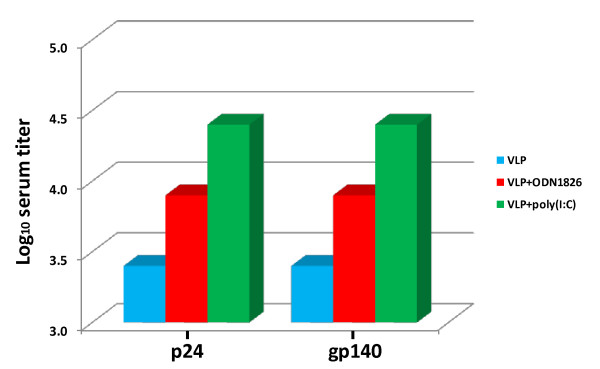
**Evaluation of anti-p24 and anti-gp140 IgG titers elicited in sera of immunized mice**. Three-fold dilutions of heat inactivated pooled mice sera, collected after the last antigen administration, were evaluated in ELISA for their reactivity with recombinant p24 and trimeric gp140. Final total IgG titers are reported in Figure as log_10 _of last dilution of sera exceeding the mean absorbance +3 standard deviations of equal dilution of pre-immunization sera.

In order to determine the effect of CpG ODN1826 and poly(I:C) adjuvants on the humoral response elicited by baculovirus-expressed HIV-VLPs developed in our laboratory, Balb/c mice were immunized by intradermal route with 20 μg of VLPs alone or formulated with the two adjuvants. Sera collected after the last boosting from all immunization groups were heat-inactivated and evaluated in ELISA against HIV-1 p24 and trimeric gp140. The results showed that both adjuvants were able to enhance the immunogenicity of HIV-VLPs eliciting a final 1:8,100 (CpG ODN1826) and 1:24,300 (poly(I:C)) titer of IgG to both p24 and gp140 (Figure 1). Such results confirm data recently reported by our Group, showing the enhanced immunogenicity of VLPs when formulated in CpG ODN1826 [81].

### Kinetic of anti HIV-1 humoral immune response

In order to determine how the anti-p24 and anti-gp140 immune responses develop in the course of the immunization protocol, sera were reacted in ELISA with recombinant gp140 or p24 after each antigen administration. In particular, sera from each group of immunized animals were pooled.

**Figure 2:**
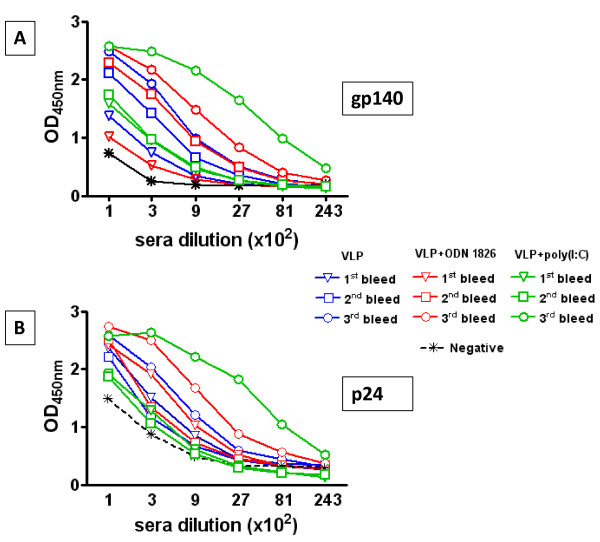
**Kinetic of anti-p24 and anti-gp140 immune responses**. Pooled sera, from each group of immunized animals, were reacted in ELISA with recombinant gp140(panel A) or p24(panel B). Total IgG titers after each antigen administration were calculated for each group. Titers were scored positive at the last dilution exceeding the mean absorbance +3 standard deviations of equal dilution of pre-immunization sera.

The results for both p24 and gp140 showed that the trend of total IgG titers is quite similar for sera from each immunization group, although those from animals immunized with poly(I:C)-adjuvanted HIV-VLPs showed a more sustained fashion (Figure 2A and 2B). Concerning the p24, the formulation of VLPs in either CpG ODN1826 or poly(I:C) induced an enhancement of total IgG titers after the third administration, compared to HIV-VLPs alone (Figure 2B). In particular, the CpG ODN1826 induced a 3-fold enhancement (1:8,100 vs 1:2,700) and the poly(I:C) induced a 9-fold enhancement (1:24,300 vs 1:2,700) (p < 0.05). Similar enhancement in the total IgG titers were observed for the gp140 (Figure 2A).

### T_H_1 polarization induced by HIV-VLPs formulated in adjuvants

The pattern of IgG subclasses induced by the different immunization protocols was evaluated by using secondary goat anti-mouse antibodies specific for each IgG subclass.

**Figure 3:**
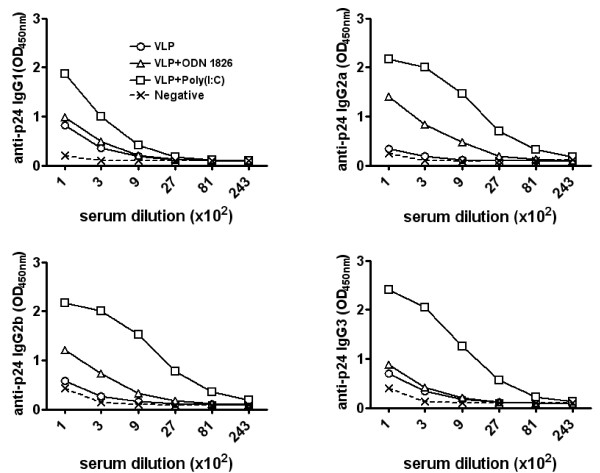
**Assessment of anti-p24 specific IgG subclasses**. Pool of sera from the last bleeding for each immunization group were reacted with recombinant p24. Anti-p24 IgG subclasses were detected with specific goat anti-mouse IgG subclasses. Titers were scored positive at the last dilution exceeding the mean absorbance +3 standard deviations of equal dilution of pre-immunization sera.

Sera from animals immunized with HIV-VLPs alone showed anti-p24 low titers (1:300) of both T_H_2 IgG1 and T_H_1 IgG3 subclasses, while no IgG2a or IgG2b were detected (Figure 3). The formulation of HIV-VLPs in CpG ODN1826 adjuvant did not enhance IgG1 and IgG3 titers, but induced a 9-fold enhancement in anti-p24 IgG2a and IgG2b titers (1:900) compare to HIV-VLPs alone (Figure 3). In contrast, HIV-VLPs formulated in poly(I:C) adjuvant induced significantly higher titers of all four IgG subclasses (Figure 3), with the highest titers observed for T_H_1 subclasses (IgG2a = IgG2b > IgG3 > IgG1).

**Figure 4:**
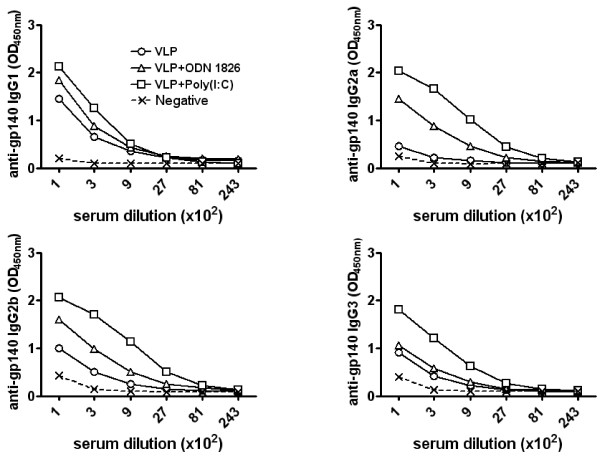
**Assessment anti-gp140 specific IgG subclasses**. Pool of sera from the last bleeding for each immunization group were reacted with recombinant gp140. Anti-gp140 IgG subclasses were detected with specific goat anti-mouse IgG subclasses. Titers were scored positive at the last dilution exceeding the mean absorbance +3 standard deviations of equal dilution of pre-immunization sera.

Similar results were observed for anti-gp140 response (Figure 4). Sera from animals immunized with HIV-VLPs alone showed medium/low titers of both T_H_2 IgG1 and T_H_1 IgG3 subclasses, while no IgG2a were detected. However, in contrast to results for anti-p24, low titers of IgG2b anti-gp140 were detected (Figure 4). The formulation of HIV-VLPs in CpG ODN1826 adjuvant induced a 3-to-9-fold enhancement in the IgG2a and IgG2b titers (1:900) compared to HIV-VLPs alone. No enhancement effects on IgG1 and IgG3 titers were observed. In contrast to results on p24, HIV-VLPs formulated in poly(I:C) adjuvant induced significantly higher titers of only T_H_1 IgG subclasses, with no enhancement of IgG1 titers (IgG2a = IgG2b > IgG3 > IgG1) (Figure 4). The analysis of titers was supported by statistical significance (p < 0.05).

Such results indicate that antibody profile induced by HIV-VLPs in mice is T_H_2 for both p24 and gp140 and that formulation in either CpG ODN1826 or poly(I:C) adjuvant results in a T_H_1 skewed IgG pattern.

### T_H_1: T_H_2 index calculation

To assess whether the adjuvant formulation elicited a specific IgG subclass profile or induced an increment in all subclasses, a T_H_1:T_H_2 index was calculated for each immunization groups. Such index takes into consideration both T_H_1 IgG2a and IgG3 subclasses compared to T_H_2 IgG1 subclass. The T_H_1:T_H_2 index showed that, in sera of animals immunized with HIV-VLPs alone, the antibody response to p24 and gp140 was T_H_2 polarized (T_H_1: T_H_2 index 0.66 and 0.22, respectively). In contrast, in sera of mice immunized with adjuvanted HIV-VLPs, the antibody response to p24 was markedly skewed towards a T_H_1 profile (T_H_1: T_H_2 index > 1), and such effect was more pronounced when HIV-VLPs were adjuvanted in poly(I:C) (T_H_1: T_H_2 index 6) (Table 1). Also for gp140, the formulation of HIV-VLPs in poly(I:C) adjuvant induced a skewing towards a T_H_1 profile (T_H_1: T_H_2 index 2) (Table 1). IgG2b titers were excluded from the index calculation because the relationship between this IgG subclass and T_H_-polarization is still debated [82,83].

**Table 1:** T_H_1:T_H_2 index for p24 and gp140

p24	VLP	VLP+ODN1826	VLP+poly(I:C)
**IgG1 (T_H_2)**	300	300	900
**IgG2a (T_H_1)**	100	900	8,100
**IgG3 (T_H_1)**	300	300	2,700
**T_H_1:T_H_2 index**	**0.66**	**2**	**6**

**gp140**	**VLP**	**VLP+ODN1826**	**VLP+poly(I:C)**

**IgG1 (T_H_2)**	900	900	900
**IgG2a (T_H_1)**	100	900	2,700
**IgG3 (T_H_1)**	300	300	900
**T_H_1:T_H_2 index**	**0.22**	**0.66**	**2**

The index has been calculated as described in Materials and Methods section. Index < 1 = T_H_2 polarization. Index > 1 = T_H_1 polarization.

## Discussion

The pattern of IgG subclass has been shown to play a role in the course of HIV infection and it has been reported to vary with progression status. In particular, a strong T_H_1 polarizing IgG subclasses as well as a broad IgG subtype response has been associated with long-term non-progressor status [28-30,84].

In order to determine the pattern of IgG subclasses induced by HIV-1 antigens packed in particulate structures as Virus Like Particles and to investigate the modulation of such pattern by T_H_1-inducing adjuvants, Balb/c mice were immunized intradermally with HIV-VLPs alone or formulated with CpG ODN1826 or poly(I:C) adjuvants.

Results shown in the present study confirmed the ability of HIV-VLPs to induce strong humoral immune response and both adjuvants were able to enhance the immunogenicity of HIV-VLPs, inducing a 3-9 fold increase in the total IgG titers for both p24 and gp140. The kinetic of induced humoral immune response showed a similar trend in sera from each immunization group, although the poly(I:C) induced a more sustained titer.

Evaluation of IgG subclasses demonstrated that HIV-VLPs alone induced anti-p24 and anti-gp140 IgG1 and IgG3 subclasses with no IgG2a elicitation. On the contrary, CpG ODN1826 and poly(I:C) showed the same capacity to enhance titers of anti-p24 and anti-gp140 IgG2a and IgG2b subclasses, compared to HIV-VLPs alone. In addition, only poly(I:C) was able to induce an enhancement also in IgG1 and IgG3 subclasses, resulting in the broadest IgG subclass pattern of immune response. Such results indicate that antibody profile induced by HIV-VLP in mice is T_H_2 for both p24 and gp140 and that formulation in both adjuvants results in a T_H_1 skewed IgG pattern. In particular, in our experimental model, poly(I:C) scores as the most potent in re-directing the T_H _pattern, in agreement to previous observations [85,86].

Such finding is further confirmed by the T_H_1:T_H_2 index which evaluates the ratio between both T_H_1 associated IgG subclasses (IgG2a and IgG3) and T_H_2 associated (IgG1) subclass. Indeed, considering that an index value > 1 stands for a T_H_1 polarization, the index in sera from animals immunized with poly(I:C)-adjuvanted HIV-VLPs scored the highest.

Our results confirm that HIV-VLPs represent an effective antigenic presentation and delivery system to prime the humoral immune response, eliciting mainly IgG1 subclass antibodies (T_H_2 profile) with other subclasses represented at low titer (IgG2b and IgG3) or not present at all (IgG2a). We demonstrated that T_H_1-inducing adjuvants, and in particular the poly(I:C), were able to broaden the IgG subclasses response and to re-direct the immune response toward a T_H_1-biased pattern. In particular, HIV-VLPs formulated in poly(I:C) adjuvant elicited the highest titer of IgG3 subclass which has been claimed to mediate the HIV neutralization in sera from HIV-infected individuals [87,88]. However all the known broadly neutralizing antibody (i.e. IgGb12, VRCO1, 2G12, 2F5 and 4E10) belong to IgG1 antibody subclass with the exception of 447 that is an IgG3 antibody. Nevertheless, crystallographic analysis of such broadly neutralizing antibodies reveal molecular structures (i.e., longer complementary determining region (CDR) H3) which suggest that these IgG1 monoclonal antibodies show properties similar to the IgG3 subclass [89-91]. These studies indicate that molecular characteristics of the IgGs (i.e., flexibility of the antibody, mobility of the F(ab) or antigen binding site) are relevant for an efficient neutralizing activity of HIV.

In this perspective, studies focused on the formulation of vaccine candidates in adjuvants able not only to enhance immunogenicity but also to re-direct IgG subclass response, may represent a key aspect in the vaccinology field. In fact, adjuvant formulations can be tailored to enhance the required immune response (antibody, cell mediated, mucosal immunity) appropriate for individual causative infectious agents [92].

## Competing interests

The authors declare that they have no competing interests.

## Authors' contributions

**MLV **performed the studies and contributed to writing the paper; **MT **contributed to HIV-VLPs preparation; **MLT **contributed to the data analysis; **FMB **supervised the whole project; **LB **was responsible for the overall planning and coordination of the study as well as writing the paper. All authors read and approved the final manuscript.
